# Cocktail supplement with rosiglitazone: a novel inducer for chicken preadipocyte differentiation *in vitro*

**DOI:** 10.1042/BSR20160049

**Published:** 2016-11-03

**Authors:** Bohan Cheng, Mengqi Wu, Songsong Xu, Xinyang Zhang, Yuxiang Wang, Ning Wang, Li Leng, Hui Li

**Affiliations:** * Key Laboratory of Chicken Genetics and Breeding, Ministry of Agriculture, Harbin 150030, PR China; † Key Laboratory of Animal Genetics, Breeding and Reproduction, Education Department of Heilongjiang Province, Harbin 150030, PR China; ‡ College of Animal Science and Technology, Northeast Agricultural University, Harbin 150030, PR China

**Keywords:** chicken, cocktail, differentiation, inducer, peroxisome proliferator-activated receptor γ (PPARγ), preadipocyte

## Abstract

The preadipocyte differentiation biological process involves a cascade of transcriptional events that culminates in the expression of peroxisome proliferator-activated receptor (PPAR) γ. The differentiation cocktail [insulin (INS), dexamethasone (DEX) and isobutylmethylxanthine (IBMX)] can induce preadipocyte differentiation in mammals, but it is insufficient for chicken (*Gallus gallus*) adipogenesis. Oleate can induce chicken preadipocyte differentiation, but these differentiated preadipocytes may not be fully functional. The objective of the current study was to evaluate whether chicken preadipocytes can be induced to mature adipocytes by a novel induction method using differentiation cocktail supplemented with PPARγ agonist(s). Chicken preadipocytes cultured in cocktail supplemented with rosiglitazone or troglitazone resulted in a marked increase in lipid droplet accumulation (*P*<0.05), glycerol-3-phosphate dehydrogenase (GPDH) activity (*P*<0.05), mRNA expression level of adipocyte fatty acid-binding protein (aP2; *P*<0.05), G_0_/G_1_ switch gene 2 (G0S2; *P*<0.05) and lipolysis (*P*<0.05). In addition, supplementation of the cocktail with rosiglitazone promoted PPARγ mRNA expression (*P*<0.05). In conclusion, our data indicated that chicken preadipocytes can be induced to mature adipocytes using differentiation cocktail supplemented with rosiglitazone. The results of the present study provide a novel induction method for *in vitro* chicken preadipocyte differentiation.

## Abstract (precis)

Chicken preadipocytes cultured in cocktail supplement with rosiglitazone resulted in a marked increase in lipid droplet accumulation, glycerol-3-phosphate dehydrogenase (GPDH) activity and mRNA expression of adipocyte fatty acid-binding protein (aP2), G_0_/G_1_ switch gene 2 (G0S2), peroxisome proliferator-activated receptor γ (PPARγ) and lipolysis. The present study provides a novel induction method for *in vitro* chicken preadipocyte differentiation.

## INTRODUCTION

The differentiation cocktail [insulin (INS), dexamethasone (DEX) and isobutylmethylxanthine (IBMX)], has been widely employed as an adipogenesis-inducing agent in mammals [1]. However, this hormone cocktail was found to be insufficient to induce chicken preadipocyte differentiation [2]. The adipogenic cocktail together with oleate [2] or oleate alone [3] could act as an inducer of chicken preadipocyte differentiation. However, studies in mouse 3T3-L1 preadipocytes showed that oleate-induced formation of fat cells with impaired INS sensitivity were metabolically distinct from adipocytes induced by hormone cocktail [4]. Therefore, we speculated that the differentiated preadipocytes induced by oleate may not be fully functional, and the method, special attention in using the cocktail to induce preadipocytes to produce functional adipocytes in chicken needs to be further investigated.

A previous study showed that the addition of GW501516 [a peroxisome proliferator-activated receptor (PPAR) β/δ agonist] to hormone cocktail did not affect adipocyte fatty acid-binding protein (aP2) expression level, and addition of troglitazone (a PPARγ agonist) to hormone cocktail slightly increased aP2 expression level, however, hormone cocktail supplemented with GW501516 and troglitazone resulted in a remarkably increased aP2 expression level and glycerol-3-phosphate dehydrogenase (GPDH) activity, but not the intracellular triacylglycerol content in chicken preadipocytes, these data indicated that PPARβ/δ does not play a key role in chicken preadipocyte differentiation [5]. Based on these results, the objective of the present study was to evaluate whether chicken preadipocytes can be induced to mature adipocytes (the increase in adipocyte-specific gene expression, the emergence of lipogenic enzyme, triacylglycerol accumulation and lipolytic capacity) by a novel induction method using hormone cocktail supplemented with a single PPARγ agonist or mixtures of PPARγ agonists.

## MATERIALS AND METHODS

### Ethics statement

All animal work was conducted according to the guidelines for the Care and Use of Experimental Animals established by the Ministry of Science and Technology of the People's Republic of China (approval number 2006-398) and approved by the Laboratory Animal Management Committee of Northeast Agricultural University.

### Primary culture of chicken preadipocytes

Chicken preadipocytes were cultured as previously described [6]. Abdominal adipose tissue was collected from 10-day-old broilers by sterile dissection following rapid decapitation. Adipose tissue was washed by pre-warmed PBS supplemented with penicillin (100 units/ml) and streptomycin (100 μg/ml; Gibco), cut with surgical scissors and digested in 2 mg/ml collagenase type I (Gibco) with shaking for 65 min at 37°C. After digestion, to separate the stromal-vascular cells from undigested tissue debris and mature adipocytes, the cell suspension was filtrated through a 100- and 20-μm mesh (BD Falcon) and centrifuged at 300 × ***g*** for 10 min at room temperature. Stromal-vascular cells (including preadipocytes) were seeded at a density of 1×10^4^ cells/ml in a basal medium [Dulbecco's modified Eagle's medium/Ham's nutrient mixture F-12 (DMEM/F12; Gibco), 10% FBS (Gibco), 100 units/ml penicillin and 100 μg/ml streptomycin (Gibco)], and maintained in a humidified atmosphere with 5% CO_2_ at 37°C.

### Induction of chicken preadipocyte differentiation

When cells reached 50% confluency, the medium for inducing differentiation was used and changed every day until day 6 of induction. Detailed inducers for the different treatments are described as in Table 1. The basal medium was prepared using DMEM/F12 containing 10% FBS, 100 units/ml penicillin and 100 μg/ml streptomycin. The hormone cocktail was composed of 20 μg/ml bovine INS (Sigma–Aldrich), 1 μM DEX (Sigma) and 0.5 mM IBMX (Sigma). The final concentrations of oleate (Sigma), rosiglitazone (Sigma), troglitazone (Sigma) and indomethacin (Sigma) were 80 μM, 5 μM, 5 μM and 0.2 mM respectively.

**Table 1:** Overview of induction medium used in the present study

Group	Induction medium
Control	Basal medium
Oleate	Basal medium + oleate
Cocktail	Basal medium + cocktail
Inducer 1	Basal medium + cocktail + rosiglitazone
Inducer 2	Basal medium + cocktail + troglitazone
Inducer 3	Basal medium + cocktail + indomethacin
Inducer 4	Basal medium + cocktail + rosiglitazone + troglitazone
Inducer 5	Basal medium + cocktail + rosiglitazone + indomethacin
Inducer 6	Basal medium + cocktail + troglitazone + indomethacin
Inducer 7	Basal medium + cocktail + rosiglitazone + troglitazone + indomethacin

### Lipid staining and measurement of lipid droplet accumulation

Lipid droplets were stained by oil red O (Sigma) according to Yagi et al. [7]. Cells cultured in 12-well plates were washed with PBS, and fixed for 30 min with 10% formalin in PBS at room temperature. Cells were rinsed again with PBS and stained with 1% oil red O staining solution (oil red O dye in 60% propan-2-ol) for 40 min at room temperature. Morphological changes of cells were observed and photographed under an inverted fluorescent microscope (Leica, Wetzlar GmbH).

Lipid droplet accumulation was measured by oil red O extraction assay [8]. After removing the staining solution, oil red O was extracted from the cells using 100% propan-2-ol and measured at 510 nm. Before lipid staining, the cell numbers in different groups were assessed based on absorbance at 450 nm using the Cell Counting Kit-8 (Dojindo Laboratory) according to the supplier's recommendations, and the cell number was used to normalize the extraction results. The effect of the inducer on lipid droplet accumulation was presented as ratio of *D*_510_/*D*_450_.

### Enzyme activity

Cells cultured in 12-well plates were washed with PBS and harvested at 1, 3 and 6 days of induction. GPDH activity was measured using the GPDH Activity Assay Kit (Takara) according to the manufacturer's instructions. Protein concentrations of cell culture homogenates were determined by the method of Lowry et al. [9] using BSA as standard.

### RNA isolation and real-time quantitative reverse transcription-PCR

Cells were harvested at 1, 3 and 6 days of induction and RNA was isolated using Trizol reagent (Invitrogen). Total RNA was quantified using an ultraviolet spectrophotometer (Eppendorf) following the manufacturer's instructions. First-strand cDNA synthesis was performed with 1 μg total RNA (Promega). To detect the expression of chicken adipogenesis-related genes, real-time quantitative reverse transcription (RT-) PCR was performed using the FastStart Universal SYBR Green Master kit (Roche Molecular Systems). A portion (1 μl) of each RT reaction product was amplified in a 10-μl PCR reaction using the ABI 7500 real-time PCR system (Applied Biosystems). PCR reaction conditions were one cycle at 95°C for 10 min and 40 cycles at 95°C for 15 s and 60°C for 1 min. Melting curves were analysed using Melting Curve 1.0 software (Applied Biosystems) for each PCR reaction to detect and eliminate possible primer–dimer artefacts. The relative expression level of target gene to β-actin was determined using the 2^−ΔCt^ method [10], in which ΔCt=Ct (target gene) − Ct (β-actin). The sequences of the primers are shown in Table 2.

**Table 2:** Primer sequences for real-time quantitative reverse transcription-PCR

Gene name	Accession number	Primer sequence (5′ to 3′)
PPARγ	AF470456	F: GTGCAATCAAAATGGAGCC
		R: CTTACAACCTTCACATGCAT
aP2	NM_204290	F: ATGTGCGACCAGTTTGT
		R: TCACCATTGATGCTGATAG
G0S2	NM_001190924	F: CTCAGCCAGAAGCCCAAC
		R: CCAACACCAAATCCTCCC
β-Actin	NM_205518	F: TCTTGGGTATGGAGTCCTG
		R: TAGAAGCATTTGCGGTGG

### Lipolysis measurement

Basal and stimulated lipolysis was assessed by measuring glycerol and non-esterified fatty acid (NEFA) release in the cell culture supernatants according to the method described by Yang et al. [11]. In brief, at day 6 of induction, cells in 12-well plates were washed with warm PBS and incubated in 1 ml of phenol red-free DMEM/F12 containing 2% fatty acid-free BSA in the presence or absence of 1 μM isoprenaline (isoproterenol, ISO). After 2 h of incubation, 50 μl of solution was removed from each sample for the measurement of glycerol or NEFA released into the medium. Commercial kits were used to analyse the amounts of glycerol (Sigma) and NEFA (Sigma) according to the manufacturer's instructions. Lysates were then prepared from the remaining cells, and protein concentrations in the lysates were used to normalize the lipolytic signals.

### Statistical analysis

Results are given as mean ± S.D. Comparison between two groups was performed by Student's *t* test. Statistical analysis among more than two groups were performed by PROC GLM procedure, followed by the Duncan's multiple range test, with the following model:

Y=μ+T+e,

in which *Y* is the phenotypic value (lipid droplet accumulation, GPDH activity, the expression level of adipogenesis-related gene, release of glycerol or NEFA), μ is the population mean, *T* is the fixed effect of treatment and *e* is the random residual effect. Differences were considered significant at *P*<0.05 unless otherwise indicated. All analyses were performed using the SAS software system (version 9.2; SAS Institute).

## RESULTS

### Effects of cocktail supplemented with PPARγ agonist(s) on lipid droplet accumulation

**Figure 1:**
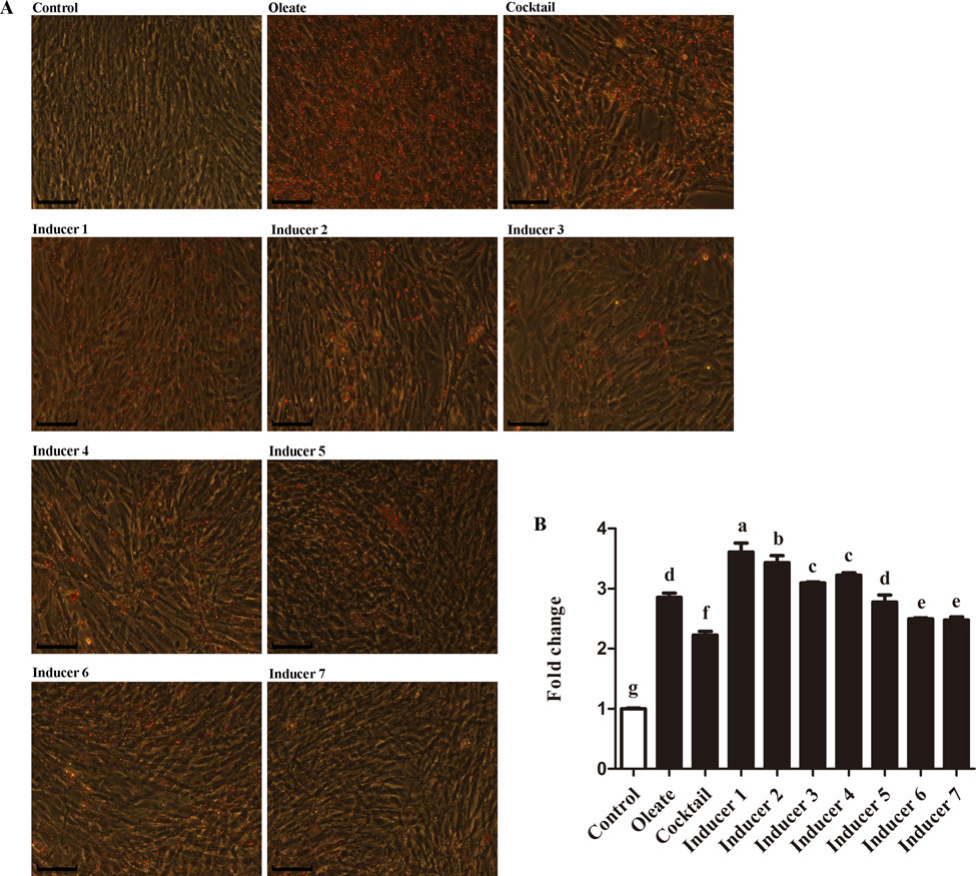
Intracellular lipid droplet accumulation in chicken preadipocytes under different treatments at day 6 of induction Intercellular lipid droplet accumulation was determined by oil red O staining and extraction assay. (**A**) Cells stained with oil red O. Scale bar, 20 μm. (**B**) Oil red O extraction assay normalized with cell number. Values are shown as the mean ± S.D. (*n*=3). ^a–g^The different lowercase letters above columns indicate significant differences among various treatments (*P*<0.05).

We first examined morphological changes of chicken primary preadipocytes under different treatments. At day 6 of induction, preadipocytes cultured in basal medium with oleate, basal medium with cocktail alone or basal medium with cocktail supplemented with PPARγ agonist(s) (Inducers 1–7) showed an increase in cytoplasmic lipid droplet accumulation compared with cells cultured in only basal medium (Figure 1A).

Lipid droplet accumulation was assessed by oil red O extraction assay at 6 days after treatments with different inducers. Chicken preadipocytes cultured in Inducers 1 or 2 (Table 1) showed significantly higher lipid droplet accumulation compared with cells cultured in basal medium, basal medium with oleate, basal medium with cocktail alone or Inducers 3–7 (Table 1) (*P*<0.05; Figure 1B). Inducer 1 showed the strongest effect on promoting lipid droplet accumulation among the inducers, and Inducer 2 showed the second strongest effect (*P*<0.05; Figure 1B).

### Effects of cocktail supplemented with PPARγ agonist(s) on GPDH activity

**Figure 2:**
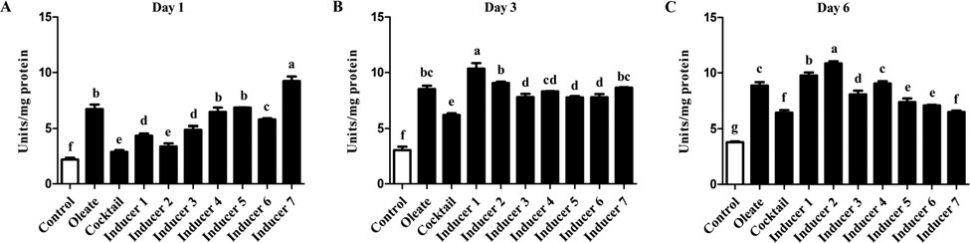
Glycerol-3-phosphate dehydrogenase activity in chicken preadipocytes under different treatments GPDH activity was measured and normalized with the total protein levels in the cell extracts. Values are shown as the mean ± S.D. (*n*=3). (**A**) Day 1. (**B**) Day 3. (**C**) Day 6. ^a–g^The different lowercase letters above columns indicate significant differences among various treatments (*P*<0.05).

At day 1 of induction, preadipocytes cultured in basal medium with oleate or Inducers 4, 5 or 7 (Table 1) showed a remarkable increase in GPDH activity compared with cells cultured in basal medium, basal medium with cocktail alone or Inducers 1, 2, 3 or 6 (Table 1; *P*<0.05; Figure 2A). At day 1, Inducer 7 showed the strongest induction of GPDH activity, whereas oleate and Inducers 4 and 5 showed the second highest GPDH induction (*P*<0.05; Figure 2A). At day 3 of induction, preadipocytes cultured in basal medium with oleate or Inducers 1, 2 or 7 showed a marked increase in GPDH activity compared with cells in basal medium, basal medium with cocktail alone or Inducers 3–6 (*P*<0.05; Figure 2B). At day 3, Inducer 1 showed the highest induction of GPDH activity, whereas oleate and Inducers 2 and 7 showed the second highest induction (*P*<0.05; Figure 2B). At day 6 of induction, preadipocytes cultured in Inducers 1 or 2 showed significantly increased GPDH activity compared with cells cultured in basal medium, basal medium with oleate, basal medium with cocktail alone or Inducers 3–7 (*P*<0.05; Figure 2C). At day 6, Inducer 2 showed the highest effect on GPDH activity, and Inducer 1 showed the second highest effect (*P*<0.05; Figure 2C).

Together these data indicated that in the early stage of chicken preadipocyte differentiation, Inducer 7 was the strongest for increasing GPDH activity in preadipocytes, and oleate and Inducers 4 and 5 were the second strongest inducers; in the middle stage of chicken preadipocyte differentiation, Inducer 1 was the strongest inducer, and oleate and Inducers 2 and 7 were the second strongest inducers and in the late stage of chicken preadipocyte differentiation, Inducer 2 was the strongest inducer and Inducer 1 was the second strongest inducer.

### Effects of cocktail supplemented with PPARγ agonist(s) on mRNA expression level of adipogenesis-related genes

**Figure 3:**
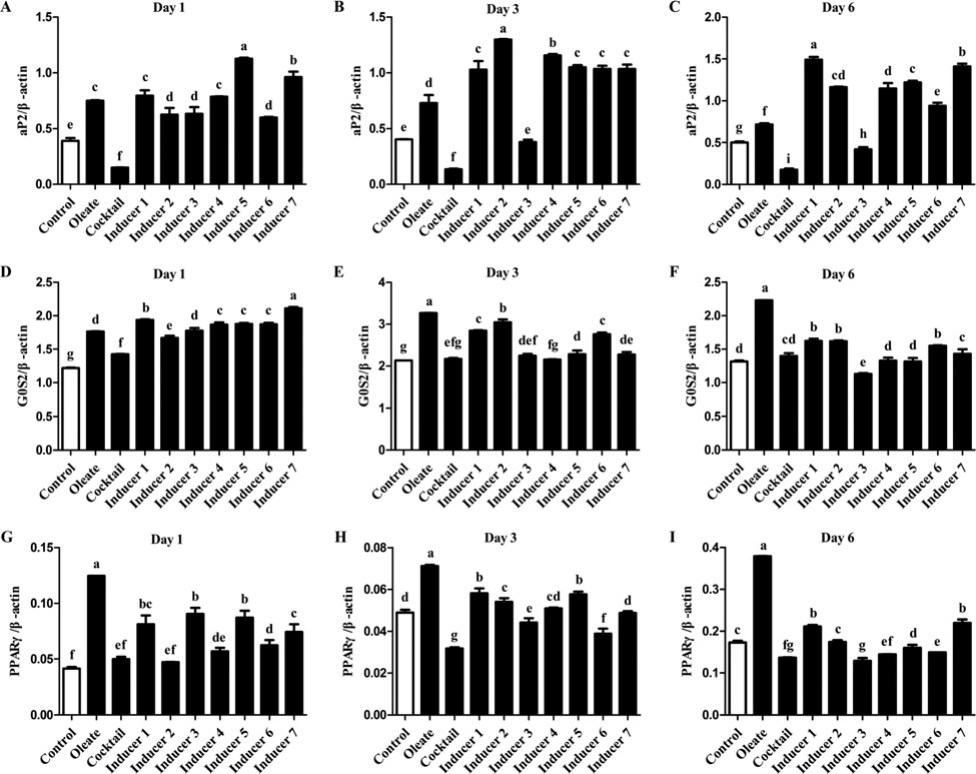
Expression level of adipogenesis-related genes in chicken preadipocytes under different treatments The expression level of each gene was determined by real-time quantitative reverse transcription-PCR. Chicken β-actin was used as the internal control. Values are shown as the mean ± S.D. (*n*=3). (**A–C**) aP2. (**D–F**) G0S2. (**G–I**) PPARγ. ^a–g^The different lowercase letters above columns indicate significant differences among various treatments (*P*<0.05).

At day 1 of induction, preadipocytes cultured in Inducers 5 or 7 (Table 1) showed a marked increase in aP2 mRNA expression levels compared with cells cultured in basal medium, basal medium with oleate, basal medium with cocktail alone or Inducers 1, 2, 3, 4 or 6 (Table 1) (*P*<0.05; Figure 3A). Inducer 5 showed a stronger promoting effect on aP2 mRNA expression levels compared with Inducer 7 (*P*<0.05; Figure 3A). At day 3 of induction, preadipocytes cultured in Inducers 2 or 4 showed significantly increased expression levels of aP2 mRNA compared with cells cultured in basal medium, basal medium with oleate, basal medium with cocktail alone or Inducers 1, 3, 5, 6 or 7 (*P*<0.05; Figure 3B). Inducer 2 showed a stronger effect on aP2 mRNA expression levels compared with Inducer 4 (*P*<0.05; Figure 3B). At day 6 of induction, Inducers 1 or 7 showed a marked increase in aP2 mRNA expression levels compared with cells cultured in basal medium, basal medium with oleate, basal medium with cocktail alone or Inducers 2–6 (*P*<0.05; Figure 3C). Inducer 1 showed a stronger effect on aP2 mRNA expression levels compared with Inducer 7 (*P*<0.05; Figure 3C).

Together these data suggested that in the early stage of chicken preadipocyte differentiation, Inducer 5 showed the strongest promoting effect on aP2 mRNA expression levels among all of the inducers and Inducer 7 showed the second strongest effect; in the middle stage, Inducer 2 was the strongest among the inducers and Inducer 4 was the second strongest inducer and in the late stage of chicken preadipocyte differentiation, Inducer 1 was the strongest among the inducers and Inducer 7 was the second strongest inducer.

At day 1 of induction, preadipocytes cultured in Inducers 1 or 7 showed significantly increased G_0_/G_1_ switch gene 2 (G0S2) mRNA expression levels compared with cells cultured in basal medium, basal medium with oleate, basal medium with cocktail alone or Inducers 2–6 (*P*<0.05; Figure 3D). Inducer 7 showed stronger effect on G0S2 mRNA expression compared with Inducer 1 (*P*<0.05; Figure 3D). At day 3 of induction, preadipocytes cultured in basal medium with oleate or Inducer 2 exhibited significantly higher expression levels of G0S2 mRNA compared with cells cultured in basal medium, basal medium with cocktail alone or Inducers 1, 3, 4, 5, 6 or 7 (*P*<0.05; Figure 3E). Oleate showed stronger effect on G0S2 mRNA expression compared with Inducer 2 (*P*<0.05; Figure 3E). At day 6 of induction, preadipocytes cultured in basal medium with oleate or Inducers 1, 2 or 6 showed a marked increase in G0S2 mRNA expression compared with cells cultured in basal medium, basal medium with cocktail alone or Inducers 3, 4, 5 or 7 (*P*<0.05; Figure 3F). Oleate showed stronger effects on G0S2 mRNA expression compared with Inducers 1, 2 and 6 (*P*<0.05; Figure 3F).

Together these results indicated that in the early stage of chicken preadipocyte differentiation, Inducer 7 showed the strongest effect on increasing G0S2 mRNA expression in preadipocytes among all of the inducers, and Inducer 1 showed the second strongest effect; in the middle stage of chicken preadipocyte differentiation, oleate was the strongest inducer and Inducer 2 was the second strongest inducer and in the late stage of chicken preadipocyte differentiation, oleate was the strongest inducer and Inducers 1, 2 and 6 were the second strongest inducers.

At day 1 of induction, preadipocytes cultured in basal medium with oleate or Inducers 1, 3 or 5 showed significantly increased PPARγ mRNA expression levels compared with cells cultured in basal medium, basal medium with cocktail alone or Inducers 2, 4, 6 or 7 (*P*<0.05; Figure 3G). Oleate showed the strongest effect on PPARγ mRNA expression among all of the inducers, and Inducers 1, 3 and 5 showed the second strongest effects (*P*<0.05; Figure 3G). At day 3 of induction, chicken preadipocytes cultured in basal medium with oleate or Inducers 1 or 5 exhibited a marked increase in PPARγ mRNA expression compared with cells cultured in basal medium, basal medium with cocktail alone or Inducers 2, 3, 4, 6 or 7 (*P*<0.05; Figure 3H). Oleate showed the strongest effect on PPARγ mRNA expression among the inducers, and Inducers 1 and 5 showed the second strongest effects (*P*<0.05; Figure 3H). At day 6 of induction, preadipocytes cultured in basal medium with oleate or Inducers 1 or 7 showed significantly increased PPARγ mRNA expression levels compared with cells cultured in basal medium, basal medium with cocktail alone or Inducers 2–6 (*P*<0.05; Figure 3I). Oleate showed the strongest effect on PPARγ mRNA expression among all of the inducers and Inducers 1 and 7 showed the second strongest effects (*P*<0.05; Figure 3I).

Together these data indicated that oleate demonstrated the strongest effect on PPARγ mRNA expression among all the inducers at every time point during chicken preadipocyte differentiation; in the early stage of chicken preadipocyte differentiation, Inducers 1, 3 and 5 showed the second strongest effects; in the middle stage of chicken preadipocyte differentiation, Inducers 1 and 5 showed the second strongest effects and in the late stage of chicken preadipocyte differentiation, Inducers 1 and 7 showed the second strongest effects.

### Effects of cocktail supplemented with PPARγ agonist(s) on lipolysis

**Figure 4:**
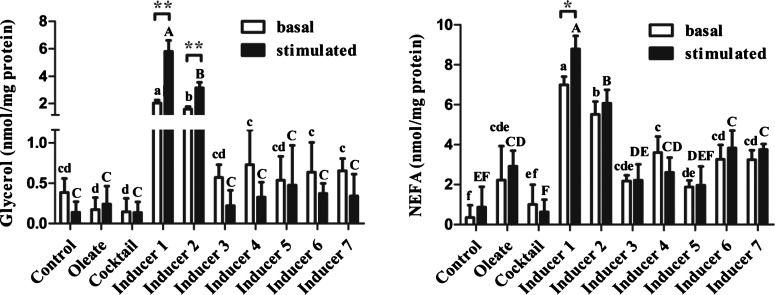
Basal and stimulated lipolysis in chicken differentiated preadipocytes under different treatments at day 6 of induction Cells were treated with (stimulated) or without (basal) 1 μM ISO for 2 h. Basal and stimulated glycerol and NEFA release were measured and normalized with the total protein levels in the cell extracts. Values are shown as the mean ± S.D. (*n*=3). Asterisks indicate significant difference between basal and stimulated condition: *P<0.05 and **P<0.01. ^a–f^The different lowercase letters above columns indicate significant differences among various treatments under basal condition (*P*<0.05). ^A–F^The different uppercase letters above columns indicate significant differences among various treatments under stimulated condition (*P*<0.05).

The effect of cocktail supplemented with PPARγ agonist(s) on lipolysis was determined by measuring glycerol and NEFA release in the cell culture supernatants. At day 6 of induction, chicken preadipocytes cultured in Inducers 1 or 2 released more glycerol and NEFA compared with cells cultured in basal medium, basal medium with oleate, basal medium with cocktail alone or Inducers 3–7 under basal and stimulated conditions (*P*<0.05; Figure 4). Inducer 1 showed the strongest lipolysis in the differentiated preadipocytes among the inducers and Inducer 2 showed the second strongest lipolytic effect (*P*<0.05; Figure 4). Cells cultured in Inducer 1 showed an increase in glycerol release (*P*<0.01; Figure 4) and NEFA release (*P*<0.05; Figure 4) under stimulated condition compared with the cells under basal condition. Similarly, cells cultured in Inducer 2 released more glycerol under stimulated condition compared with those of under basal condition (*P*<0.01; Figure 4).

## DISCUSSION

The increase in adipose tissue mass is due to an increase in the number and size of adipocytes [12]. Adipocyte number is regulated by the commitment of mesenchymal stem cells to the adipocyte lineage and by the proliferation of preadipocytes, whereas adipocyte size is regulated by the differentiation of preadipocytes [13]. The biological process of preadipocyte differentiation has been extensively studied in mammalian preadipocyte cell lines, such as mouse 3T3-L1 and 3T3-F442A cells [7,14]. These studies have revealed a complicated regulatory network of transcription factors that underlies preadipocyte differentiation [1,15–18]. PPARγ is the most important transcription factor during adipogenesis, which involves the induction and stimulation of fat-specific genes, including aP2, G0S2 and GPDH in mammals [19,20]. We previously reported that PPARγ is a key regulator of preadipocyte differentiation in chickens [21]. PPARγ agonists, such as thiazolidinediones (TZDs), mediate the regulation of preadipocyte differentiation and lipid metabolism [22–24]. As for the molecular mechanism that controls adipogenesis, previous studies indicated that species-specific differences exist between mammals and birds [25]. A better understanding of the physiological characteristics of and molecular events in chicken preadipocyte differentiation will not only broaden our knowledge of adipocyte development across species, but also provide clues for reducing excessive fat deposition in chickens, which is one of the major complications in the modern broiler industry [26,27].

Although chicken preadipocytes can be induced to differentiate into adipocytes by adding oleate [2,3], the fat cells induced by oleate may not be fully functional [4]. The differentiation cocktail (INS, DEX and IBMX) can induce preadipocyte differentiation in mammals, but it is insufficient for chicken adipogenesis [2]. An induction method using differentiation cocktail supplemented with troglitazone (a PPARγ agonist) and GW501516 (a PPARβ/δ agonist) developed by Sato et al. [5] increased aP2 expression and GPDH activity but not lipid droplet accumulation in chicken preadipocytes. In view of this, the present study was designed to explore a novel induction method using differentiation cocktail supplemented with a single PPARγ agonist (troglitazone, rosiglitazone or indomethacin) or mixtures of PPARγ agonists for chicken preadipocyte differentiation *in vitro*. DEX is recognized as an essential inducer of preadipocyte differentiation for *in vitro* studies of both cell lines and primary culture [28,29]. A previous study showed that IBMX acts as an inducer of cAMP and plays a key role in adipogenesis [1]. Therefore, in our experiments, we treated cells with a combination of DEX, IBMX and INS during the whole induction, and this method was different from previously published experiments by Sato et al. [5]. In their experiments, chicken preadipocytes were cultured in a similar manner to mouse 3T3-L1 cells [30], that cocktail being switched to INS alone after 48 h of induction. In the present study, at day 6 of induction, Inducers 1 and 2 showed the strongest promoting effect on cytoplasmic lipid droplet accumulation compared with other inducers. These results indicated that Inducers 1 and 2 are better than other inducers for *in vitro* fat accumulation in chickens. This observation was not consistent with results obtained in chicken preadipocytes induced by cocktail supplemented with troglitazone and GW501516, in which lipid droplet accumulation was not increased at day 7 of induction [5]. Thus, the difference in lipid droplet accumulation in chicken preadipocytes between the study by Sato et al. [5] and the current study may be attributed to the difference of cocktail components during induction.

The final product of adipogenesis is a functional adipocyte and this mature cell is characterized by the increase in adipocyte-specific gene expression level, the emergence of lipogenic enzyme, triacylglycerol accumulation and lipolytic capacity [31–34]. To determine whether differentiated preadipocytes induced by cocktail supplemented with PPARγ agonist(s) are functional, we examined markers of metabolic functions of adipocytes, such as GPDH activity, expression levels of aP2, G0S2 and PPARγ, glycerol and NEFA release. At day 1 of induction, the promoting effects of oleate and Inducers 4, 5 and 7 on GPDH activity were stronger than other inducers; at day 3 of induction, oleate and Inducers 1, 2 and 7 were stronger than other inducers and at day 6 of induction, Inducers 1 and 2 were stronger than other inducers. In general, chicken preadipocytes need 5–7 days differentiation *in vitro* [2,3], and as GPDH is a late marker of preadipocyte differentiation [31], our data show that Inducers 1 and 2 are more effective at inducing chicken preadipocyte differentiation *in vitro* compared with other inducers.

The aP2 protein serves as an intracellular binding partner for long-chain fatty acids [35], and it can also function in ligand-dependent transactivation of PPARγ by trafficking long-chain fatty acids (such as oleate) to the nucleus [36]. In addition, aP2 is a marker gene of preadipocyte differentiation [15,37,38]. In the current study, at day 1 of induction, Inducers 5 and 7 showed the strongest effect on aP2 mRNA expression; at day 3 of induction, Inducers 2 and 4 were the strongest inducers and at day 6 of induction, Inducers 1 and 7 were the strongest inducers. As aP2 is a late marker of preadipocyte differentiation [39], our data show that Inducers 1 and 7 are more effective at inducing chicken preadipocyte differentiation *in vitro* compared with other inducers.

Previous studies have shown that G0S2 inhibits lipolysis by attenuating adipose triacylglycerol lipase [11,40]. A recent study demonstrated that G0S2 has a critical role in preadipocyte differentiation and accumulation of triacylglycerol [41]. In the current study, at day 1 of induction, Inducers 1 and 7 showed the strongest effect on G0S2 mRNA expression; at day 3 of induction, oleate and Inducer 2 were the strongest inducers and at day 6 of induction, oleate and Inducers 1, 2 and 6 were the strongest inducers. As G0S2 is a late marker of preadipocyte differentiation [11], our results show that oleate and Inducers 1, 2 and 6 are better inducers for inducing chicken preadipocyte differentiation *in vitro* compared with other inducers.

Previous studies showed that PPARγ is a master regulator of chicken preadipocyte differentiation [2,21], and chicken embryo fibroblasts can be induced to transdifferentiate into adipocyte-like cells by the ectopic expression of PPARγ via retrovirus-mediated gene transfer [42]. At days 1, 3 and 6 of induction, our data showed high mRNA expression level of PPARγ after oleate treatment, which is consistent with previous reports in chickens that showed that oleate enhanced PPARγ gene expression [2,3]. This phenomenon was also observed in the current study in response to Inducer 1. Taken together, these data suggested that Inducer 1 may promote chicken preadipocyte differentiation, at least in part, by activating the molecular programme controlled by PPARγ. Interestingly, except for oleate and Inducer 1, other inducers did not increase PPARγ mRNA expression at each time point during the chicken preadipocyte differentiation process. As activation of PPARγ expression is essential for chicken preadipocyte differentiation [21], oleate and Inducer 1 are better for inducing chicken preadipocyte differentiation *in vitro* compared with other inducers. Notably, Inducers 1, 4, 5 and 7 all contain rosiglitazone. The reason underlying the stronger adipogenic effect of Inducer 1 on chicken preadipocytes than Inducers 4, 5 or 7 is not clear, though there may be unknown interactions between different PPARγ agonists.

Lipolysis is complicated and is related to multiple genes and pathways [43,44]. In the present study, at day 6 of induction, Inducers 1 and 2 showed the strongest lipolysis in the differentiated preadipocytes compared with other inducers. This observation is consistent with those obtained in mouse adipocytes, in which rosiglitazone and troglitazone markedly increased lipolysis [45,46]. It is worth noting that ISO-stimulated lipolysis in chicken differentiated preadipocytes was enhanced compared with basal lipolysis. A previous study showed that lipolysis could be stimulated by a number of agonists, such as ISO, forskolin, dibutyryl cyclic AMP, cilostamide and rolipram in mammals [47]. Therefore, our results show that chicken differentiated preadipocytes are also sensitive to ISO *in vitro*. Considering adipocytes could perform their function of lipid metabolism, our data indicate that Inducers 1 and 2 are more effective at inducing chicken preadipocytes to produce functional adipocytes compared with other inducers.

The transcriptional activity of PPARγ is induced by a variety of ligands [48,49]. Rosiglitazone is a well-known PPARγ agonist that increases peripheral INS sensitivity and improves glycaemic control in type 2 diabetes [50]. Furthermore, rosiglitazone induced preadipocyte differentiation in cell culture models and increased weight gain in mammals [51]. Recent research showed that DEX and rosiglitazone are sufficient and necessary for producing functional adipocytes from mesenchymal stem cells [33]. Troglitazone is a member of the drug class of TZDs, known as specific anti-diabetic agents that decrease plasma glucose levels and enhance INS sensitivity in obese mammals [52]. Troglitazone alone or in combination with DEX induced porcine preadipocyte differentiation together with an increase in PPARγ expression, but with little lipid accretion [53]. A previous study reported that the administration of troglitazone to growing chickens increased abdominal adipose mass [24]. Indomethacin is a PPARγ ligand that is clinically used for its anti-inflammatory, anti-pyretic and analgesic properties [54]. Previous studies showed that indomethacin can promote terminal differentiation of mouse 3T3-L1 preadipocytes [55–57]. In the current study, chicken preadipocytes cultured in cocktail supplemented with rosiglitazone (Inducer 1) or troglitazone (Inducer 2) showed a remarkable increase in the accumulation of lipid droplet, GPDH activity and mRNA expression of aP2 and G0S2 compared with cells cultured in cocktail supplemented with indomethacin (Inducer 3). These data indicated that the adipogenic role of rosiglitazone or troglitazone may be stronger than that of indomethacin in chicken preadipocytes.

Fatty acids are ubiquitous biological molecules. In addition to providing energy and constituting cell membrane to regulate physiological functions of cells, they can function as mediators of signal transduction and transcription for many genes [58,59]. As an important member of fatty acids, oleate is essential for preadipocyte differentiation in chickens [2,3]. The results of the current study indicated that Inducer 1 and oleate are better inducers for chicken preadipocyte differentiation compared with other inducers. Chicken preadipocytes cultured in Inducer 1 showed a significant increase in lipid droplet accumulation (1.26-fold), GPDH activity (day 6, 1.10-fold), mRNA expression levels of aP2 (day 6, 2.08-fold), glycerol release (basal condition, 11.87-fold; stimulated condition, 24.00-fold) and NEFA release (basal condition, 3.14-fold; stimulated condition, 3.01-fold) compared with cells cultured in basal medium with oleate. Meanwhile, preadipocytes cultured in basal medium with oleate showed remarkably higher mRNA expression levels of G0S2 (day 6, 1.38-fold) and PPARγ (day 1, 1.54-fold; day 3, 1.22-fold; day 6, 1.80-fold) compared with cells cultured in Inducer 1. It may be relevant to consider that fatty acids have a direct and potent action to increase PPARγ mRNA expression [2,3,60]. Nonetheless, chicken differentiated preadipocytes induced by Inducer 1 may possess the stronger function compared with the cells induced by oleate, because one of the main characteristics of a functional adipocyte is its lipolytic capacity [34]. Taken together, Inducer 1 (cocktail supplement with rosiglitazone) is likely to be a better inducer for inducing chicken preadipocyte differentiation *in vitro* compared with oleate.

In summary, although further studies are required to elucidate the molecular mechanisms underlying the adipogenic effect of cocktail supplement with rosiglitazone on chicken preadipocytes, our findings clearly demonstrate that hormone cocktail supplement with rosiglitazone can act as a novel inducer for *in vitro* chicken preadipocyte differentiation.

## Abbreviations

aP2: adipocyte fatty acid-binding protein
DEX: dexamethasone
DMEM/F12: Dulbecco's modified Eagle's medium/Ham's nutrient mixture F-12
G0S2: G_0_/G_1_ switch gene 2
GPDH: glycerol-3-phosphate dehydrogenase
IBMX: isobutylmethylxanthine
INS: insulin
ISO: isoprenaline
NEFA: non-esterified fatty acid
PPAR: peroxisome proliferator-activated receptor
TZD: thiazolidinedione
